# Upcycling clarified and decolorized red beet waste into a sustainable glucose syrup alternative for ice cream production

**DOI:** 10.1016/j.fochx.2026.104073

**Published:** 2026-06-06

**Authors:** Tahra ElObeid, İlyas Atalar, Burcu Tüzün, Ibrahim Palabiyik, Omer Said Toker, Nevzat Konar

**Affiliations:** a Qatar University, QU Health, College of Health Sciences, Department of Nutrition Sciences, Doha, Qatar; b Eskisehir Osmangazi University, Agriculture Faculty, Food Engineering Department, Eskisehir, Türkiye; c Tekirdag Namik Kemal University, Agriculture Faculty, Food Engineering Department, Tekirdag, Türkiye; d Yıldız Technical University, Faculty of Chemical and Metallurgical Engineering, Food Engineering Department, İstanbul, Türkiye; e Ankara University Agriculture Faculty, Dairy Technology Department, Ankara, Türkiye

**Keywords:** Ice cream, Waste treatment, Red beet, Glucose syrup, Sugar substitute

## Abstract

This study aimed to determine the potential use of the liquid waste (RBLW, ∼12.0°Bx) generated after pigment extraction from industrial red beet (RB) (*Beta vulgaris* L.) juice concentrate during the production of RB extract powder as an alternative to glucose syrup in ice cream technology. After clarification and decolorization of RBLW, the effects of its reduced-sugar profile, high ash content, low pH, and residual pigments on the visual properties of ice cream, which was used as a model food, were determined. Moreover, the effects of ash content and pH on milk proteins were examined. At the initial stage of the study, clarification and decolorization processes were carried out on RBLW using anion and cation exchange resins and an adsorber resin (modified styrene-DVB), resulting in a clarity (T625) value of 77.3%, and the solution was evaporated to >65°Bx. Ice cream samples were prepared using an RSM custom mixture design, with upcycled RBLW, glucose syrup, and stabilizer (salep) as independent variables (*n* = 14). Significant models were identified with R^2^ values ranging from 0.60199 to 0.9212 for the viscosity, consistency, flow index, pH, ash content, a*, b*, chroma, and hue angle properties of ice cream mixes, and from 0.6614 to 0.9790 for the a*, hue angle, hardness, and melting rate properties of ice cream samples. The results indicated that RBLW, after clarification and decolorization, could be used in ice cream and that the waste from the natural colorant industry can be utilized as innovative food ingredients within the scope of the circular economy.

## Highlights

• Clarified red beet waste was used as a glucose syrup alternative in ice cream.
• Resin treatment improved clarity and functionality of red beet liquid waste.
• RBLW influenced ice cream texture, color, and melting behavior effectively.
• RSM modeling showed high significance for mix and product quality traits.
• Study supports upcycling of food waste for novel ingredient development.

## Graphical abstract

**Figure:**
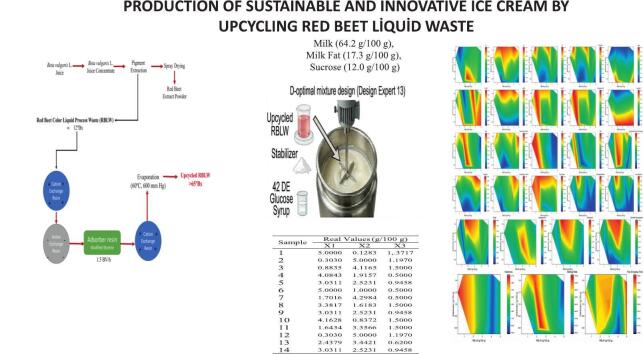

## 1 Introduction

As in other food technology fields, sugar substitution studies are important within the scope of dairy technology (McCain et al., 2018). Among these products, ice cream is characterized by its high sugar content (Biguzzi et al., 2014). However, the sugars in ice cream composition are not only related to sensory properties. They also interact with various other quality characteristics. For example, sugar's crystallization behavior and tendencies affect textural properties (Abbasi & Saeedabadian, 2015; Skryplonek et al., 2017). However, to achieve the sweet flavors expected by consumers, approximately 10–14% sugar usage is required (Goff, 2015). The main sugars used for this purpose are sucrose and glucose syrup (Acan et al., 2020). Additionally, lactose, a sugar in ice cream composition, affects product quality, particularly through its crystallization behavior (McCain et al., 2018). Changes in sugar composition and concentration affect the perception intensity or masking of some flavor and taste components in ice cream (Harwood et al., 2013). Furthermore, fat reduction or substitution, frequently used in low-calorie ice cream development studies, is associated with product behavior in the oral cavity and perceived sweetness and flavor intensity (Karaca et al., 2009). There are two main motivations in studies on sugar substitution in ice cream composition: (i) the use of alternative low-calorie sweeteners to develop low-calorie and/or sugar-free formulations, or (ii) evaluating the potential use of alternative materials developed through recovery (or recycling) to replace sugars (sucrose and/or glucose syrup) in product composition. The most frequently used of these is ice cream reformulation with low- or negligible-calorie components to address its high calorie content. In this context, various components have been used, such as maltodextrin (Rolon et al., 2017), stevia (Ahmed et al., 2023), inulin (Akin et al., 2007; da Silva Faresin et al., 2022), erythritol (Moriano & Alamprese, 2017), and different polyol mixtures (Kalicka et al., 2019). In using innovative bulking agents, the focus has been on utilizing waste and/or by-products, particularly as sugar substitutes. Coconut milk residue (Hanafi et al., 2022), herbal extracts and fructooligosaccharides (Gremski et al., 2019), agave fructans (Jardines et al., 2020), fruit (grape, apricot, apple) and cereal (rice, corn, sunflower, barley) dietary fibers (Ayar et al., 2018), licorice extract (Nooshkam et al., 2023), persimmon (Karaman et al., 2014), chia seeds (Velotto et al., 2021), whey protein concentrate (Schweiger et al., 2023), spray-dried sugar beet molasses (Acan et al., 2020) can be given as example. However, no study has been conducted using red beet processing waste in ice cream composition. In the food industry, processing waste poses significant global economic, ecological, and social challenges due to the sheer volume generated and its complex composition (Martin-Rios et al., 2018). Consequently, recycling and reuse practices are prioritized within environmental and food security policies (Corrado & Sala, 2018). According to UN Sustainable Development Goal 12, food waste should be reduced by at least half by 2030, necessitating urgent implementation of recovery activities throughout the supply chain. A particularly beneficial approach involves upcycling waste generated during food processing back into the food industry itself (Pleissner, 2018). Red beet extract is a widely used natural colorant; however, its industrial production is characterized by a low-efficiency process that generates substantial volumes of liquid by-products containing various macronutrients and micronutrients, as well as residual pigments (Atalar et al., 2024). The persistence of these pigments in the secondary stream complicates the direct reuse and conventional treatment processes of these materials (Costa et al., 2017). Consequently, establishing viable reuse strategies through targeted pre-treatments is essential. The current literature remains limited, often focusing on the high-energy drying of liquid by-products or on evaluating solid pulp as a secondary pigment source (Atalar et al., 2024; Costa et al., 2017). The substantial energy demand of such drying processes represents a significant technological disadvantage. Alternatively, the recovery of macromolecules (e.g., proteins) and micromolecules (e.g., glucose, fructose, sucrose) from these liquid process streams offers a more sustainable pathway. Since red beet juice is naturally rich in sugars, the resulting processing by-products maintain a high sugar concentration (Atalar et al., 2024). While this high organic load presents a challenge for waste disposal, it significantly enhances the material's potential for upcycling. Specifically, the protein content of this liquid stream is approximately 10.0 g/100 g (dry matter), while the total concentration of monosaccharides (glucose, fructose) and disaccharides (sucrose) exceeds 70.0 g/100 g (dry matter). Although this composition shows strong potential as a substitute for conventional corn syrups, the presence of impurities, particularly residual betalain pigments, necessitates specialized pretreatment processes to ensure functional and visual compatibility with food systems.

Betalains, the primary pigments in red beet, exhibit significant bioactive potential due to their antioxidant and antimicrobial properties. However, the persistence of these pigments as residues in the process liquid stream significantly restricts the versatility and industrial application of the resulting by-products. In the production of sugar syrups, visual attributes, specifically color and turbidity, serve as paramount indicators of quality and overall sensory appeal (Henke et al., 2019). Consequently, efficient color removal is essential for stabilizing these syrups. The most prevalent method for this purpose in the sugar and fruit juice industries involves the integrated use of ion-exchange and adsorbent resins. Effective pigment removal depends on several critical factors, including the molecular weight, polarity, and degree of ionization of the target molecules, the latter of which is fundamentally influenced by the pH of the medium (Li et al., 2019). Successful decolorization not only broadens the range of utilization for these syrups but also prevents visual stability issues and eliminates undesirable chemical interactions. Building upon this technological framework, the present study aims to evaluate the potential of clarified and decolorized Red Beet Liquid Waste (RBLW) obtained through anion, cation, and adsorbent resin treatments as a sustainable alternative to conventional glucose syrup in ice cream formulations. The primary motivation for using upcycled RBLW is its favorable reducing-sugar profile, particularly its high glucose and fructose concentrations. Ice cream was selected as the model food system for two strategic reasons: (i) its inherent visual properties allow for a precise assessment of the effectiveness of the decolorization process and the impact of any residual pigments, and (ii) its complex dairy matrix, characterized by a higher ash content compared to standard glucose syrup, provides an ideal environment to observe the upcycled RBLW's influence on the stability and interactions of milk proteins.

## 2 Materials and methods

### 2.1 Materials

In the preparation of ice cream samples, sucrose (Konya Sugar, Konya, Turkey), 40–42 DE glucose syrup (Sunar, Adana, Turkey), milk fat, cow milk (Halk, Eskisehir, Turkey) and salep (Tayas, Kocaeli, Turkey) as stabilizer were used. Red Beet Colorant Process Liquid Waste (RBLW) was obtained from an industrial coloring agent producer (X Company, Kocaeli, Turkey).

### 2.2 RBLW preparation and characterization

The RBLW used in this study was obtained from an industrial producer (X Company, Kocaeli, Turkey) as a liquid waste byproduct of red beet extract powder production. This material (∼12.0°Bx soluble solid content, 20.0 ± 1.00 °C, pH < 3.50) is a by-product after the extraction of pigments from juice concentrate. For clarification and decolorization of RBLW, the samples were transferred to columns with a 4 cm inner diameter. The RBLW was first passed through a cation exchange resin (Macro-Prep 25S, Bio-Rad, Turkey) at a flow rate of 2 bed volumes per hour (BV/h), and after the pH value of RBLW was adjusted to 8.00 ± 0.20 by 1 M NaOH, it was passed through an anion exchange resin (Macro-Prep, High Q, Bio-Rad, Turkey) at the same rate. Then, the samples (<3.5 pH) were decolorized by using an adsorber resin. For this aim, the flow rates were set at 1.50 and 2.00 BV/h, and the adsorber used was Modified Styrene-DVB (600.0 m^2^/g, 1.18 g/mL). The samples were then passed through the cation exchange resin (Macro-Prep 25S, Bio-Rad, Turkey) again at a flow rate of 2 BV/h. The clarified and decolorized samples were evaporated (60 °C, 600 mmHg vacuum, pilot-type triple-effect falling-film evaporator, X Company, Kocaeli, Turkey) until they reached a minimum soluble content of 65.0°Bx. The obtained RBLW samples were stored in sterilized glass containers (121 °C for 15 min) at 4 °C in a dark environment until analysis.

The pH value of RBLW samples was measured using a digital pH meter (PB-10, Sartorius, Germany) calibrated with buffers at pH 4.00, 7.00, and 10.00. In preliminary studies, this measurement was performed on all sample groups before and after each resin application and evaporation. The pH values were determined after evaporation in the deionization and decolorization studies. The total solid content of the samples after evaporation was determined in Brix (°Bx) using a hand-held automatic digital refractometer (PAL, Atago, Japan). The ash content of the samples before and after evaporation was determined as a percentage (% m/m) based on the inorganic residue remaining after complete combustion. For this purpose, 5 g of the sample was placed in a pre-weighed, dried crucible and heated in an ash furnace at 500 °C for 3 h. After cooling in a desiccator, the samples were weighed to determine the ash content (AOAC, 2005). The optical properties of the Red Beet Process By-product (RBPB) were monitored to assess the efficiency of the resin treatments. Clarity was quantitatively defined as the percent transmittance at 625 nm (T625) (Zhao et al. (2014). Since the evaporation process produced a concentrated syrup (>65.0°Bx), direct spectrophotometric measurement was not feasible due to the high optical density. Therefore, after evaporation, the samples were re-dissolved in distilled water to their initial concentration (∼12.0°Bx) prior to analysis. Absorbance and transmittance were measured at 625 nm and 420 nm, respectively, using a UV–Vis spectrophotometer (UV-1240, Shimadzu, Kyoto, Japan). Additionally, the visual profile was characterized using CIELAB color parameters (L*, a*, b*, chroma, and hue angle) measured with a colorimeter (Chroma Meter CR-400, Konica Minolta, Japan).

Crude protein content (Nx6.25) was determined using the Kjeldahl Method described by Ziena (2000). Total acidity was determined using 0.2 M sodium hydroxide solution and phenolphthalein as an indicator, and the results were expressed as grams of citric acid equivalent per liter (g citric acid/L) (Lisiewska & Kmiecik, 2000). Total sugar content was determined using Fehling's solutions via the titrimetric method. After evaporation, the sugar profile (sucrose, fructose, glucose contents) of the RBLW samples was determined using an HPLC system equipped with a refractive index detector. For this purpose, a 55 °C Aminex HPX-87H column (300 × 7.8 mm) (Bio-Rad, Istanbul) was used, with a flow rate of 0.3 mL/min and a mobile phase consisting of 6% acetonitrile and 0.045 N H_2_SO_4_ (Kelebek et al., 2009). The values of LOQ: % 0.10 for fructose, 0.15 for glucose, 0.5 for sucrose; The values of LOD: % 0.03 for fructose, 0.05 for glucose, 0.17 for sucrose. The antioxidant activity potential of the samples was determined using the DPPH method based on the % inhibition values (Demirci et al., 2024). For the total phenolic content (TPC) analysis, 40 μL of the sample, diluted with methanol, was placed into a spectrophotometer cuvette (macro), and 3.16 mL of distilled water and 200 μL of Folin-Ciocalteu reagent solution (Merck, Darmstadt, Germany) were added. After waiting 1–2 min, 600 μL of 20% sodium carbonate (Merck, Germany) solution was added, and the mixture was left at room temperature for 2 h before measuring absorbance at 765 nm with a spectrophotometer (UV-1240, Shimadzu, Kyoto, Japan) against a blank. The total phenolic content was calculated using a gallic acid calibration curve and expressed in milligrams of Gallic Acid Equivalent (GAE) per kilogram (Atalar et al., 2024). Results of clarified and decolorized RBLW analysis are given in Table 1. (See Table 2.)

**Table 1:** Composition and Characteristic of Clarified and Decolorized Red Beet Colorant Process Liquid Waste (RBLW).

Parameter	Clarified and Decolorized RBLW
Transmittance 625 nm^a^ (%)	77.3 ± 3.58
Total soluble solids (°Bx)	66.3 ± 0.23
pH	3.32 ± 0.01
Total acidity (g citric acid/L)	0.63 ± 0.03
L*	76.6 ± 2.32
a*	−1.71 ± 0.90
b*	52.9 ± 7.12
Chroma	52.8 ± 7.10
Hue angle	91.9 ± 1.07
Browning index (A420 nm)	1.27 ± 0.03
Total phenolic content (mg GAE/kg)	58.1 ± 0.83
Antioxidant activity potential^b^ (%, inhibition)	20.2 ± 0.01
Crude protein (g/100 g)	3.10 ± 0.41
Total ash (g/100 g)	5.05 ± 0.01
Total sugar (g/100 g)	65.21 ± 2.35
Sucrose (g/100 g)	12.5 ± 1.04
Glucose (g/100 g)	24.8 ± 3.64
Fructose (g/100 g)	25.3 ± 1.87

^a^The degree of clarification, ^b^ by DPPH method, GAE; gallic acid equivalent, mean ± standard deviation.

**Table 2:** D-optimal mixture design study model and sample preparation (g/100 g).^a^

Sample	Real Values			Milk	Sucrose	Milk Fat
	X1	X2	X3			
1	5.00	0.13	1.37	64.2	12.0	17.3
2	0.30	5.00	1.20	64.2	12.0	17.3
3	0.88	4.12	1.50	64.2	12.0	17.3
4	4.08	1.92	0.50	64.2	12.0	17.3
5	3.03	2.52	0.95	64.2	12.0	17.3
6	5.00	1.00	0.50	64.2	12.0	17.3
7	1.70	4.30	0.50	64.2	12.0	17.3
8	3.38	1.62	1.50	64.2	12.0	17.3
9	3.03	2.52	0.95	64.2	12.0	17.3
10	4.16	0.84	1.50	64.2	12.0	17.3
11	1.64	3.36	1.50	64.2	12.0	17.3
12	0.30	5.00	1.20	64.2	12.0	17.3
13	2.44	3.44	0.62	64.2	12.0	17.3
14	3.03	2.52	0.95	64.2	12.0	17.3

a ; g/100 g. X_1_. X_2_. and X_3_. Clarified and Decolorized RBLW (g/100 g). Glucose Syrup (42 DE) (g/100 g) and Stabilizer (g/ 100 g) respectively. RBLW; Red Beet Liquid Waste.

### 2.3 Study design and ice cream production

The study design was prepared using the D-Optimal Mixture Design of the Response Surface Method (Design Expert, trial ver. 13). The independent variables were clarified and decolorized RBLW (0.00–5.00 g/100 g), glucose syrup (0.00–5.00 g/100 g), and stabilizer (0.50–1.50 g/100 g). To ensure consistency, the amounts of sucrose, milk fat, and milk were kept constant at 12.0, 17.3, and 64.2 g/100 g, respectively. During production, the mixture was first pre-heated to 80 °C and homogenized at 10,000 rpm for 2 min using a high-speed disperser  (T25 Ultra-turrax IKA-Werke GmbH & Co. KG, Staufen, Germany). Pasteurization was then completed in a water bath at 80 °C for 10 min. Following heat treatment, samples were rapidly cooled to 4 °C and aged for 24 h at this temperature. Ice creams were manufactured by whipping 500 g of the aged frozen mixes in a batch ice cream maker (Delonghi, ICK 5000, China) without additional aeration, and all samples were produced in triplicate to ensure statistical reliability.

### 2.4 Ice cream mix analysis

#### 2.4.1 Physico-chemical analysis

The dry matter (DM) content was determined gravimetrically by drying a known weight of the sample in a forced-air oven at 105 °C until a constant weight was achieved. The ash content was measured by incinerating the samples in a muffle furnace at 550 °C for approximately 5 h until a light gray or white ash remained, representing the total inorganic residue. For pH measurements, a digital pH meter equipped with a temperature-compensated electrode was used. The device was calibrated using standard buffer solutions at pH 4.0, 7.0, and 10.0, and readings were taken directly from the aged mixes at 20 °C(Atalar et al., 2021).

#### 2.4.2 Color analysis

The surface color of the gummy candies (L*: brightness, a* ± red–green, b*:±yellow–blue,) was determined using a colorimeter device (Chroma Meter CR-400, Konica Minolta, Japan). Chroma and hue angle were calculated using Eq. 1 and Eq. 2 below;

(1) $$C^{*}=\sqrt{{a^{*}}^{2}+{b^{*}}^{2}}$$

(2) h°=arctanb∗a∗

#### 2.4.3 Rheology

Rheometric measurements of the ice cream mixes aged for 1 day at 4 °C were performed using a rheometer (Haake Mars 40 model, Thermo Scientific, Darmstadt, Germany) equipped with parallel plate geometry (diameter: 35 mm, gap: 1 mm). All measurements were conducted at 4 °C. The samples were analyzed at a shear rate of 0–100 s^−1^ for 2 min for steady shear rheological properties. The samples' flow behavior and consistency index were calculated using the Ostwald-de-Waele model (Eq. 3).

(3) $$\eta_{\mathrm{app}}=Ky^{n-1}$$

where η_app_ is the apparent viscosity (Pa.s); K is the consistency index (Pa.s^n^); γ̇ is the shear rate (s^−1^) and n is the flow behavior index. Model calculations were performed with Rheowin 4 Software v4.20 (Haake Company, Darmstadt, Germany). The results are reported in Table 3 as mean ± standard deviation (*n* = 3). Frequent sweep tests were conducted under a force of 0.01 Pa at frequency ranges of 0.1–10 Hz to determine the dynamic shear rheological properties. Stress sweep tests were applied to determine the linear viscoelastic range for measuring the storage modulus (G′) and loss modulus (G′′).

**Table 3:** Physicochemical, Rheological and Color Properties of Ice Cream Mix.

Sample	DM (%)	AC (g/100 g)	pH	η_app_	K	n	R^2^	G' (Pa)	G" (Pa)	L*	a*	b*	Chroma	Hue Angle
1	30.1 ± 0.06	0.66 ± 0.01	6.71 ± 0.01	0.37 ± 0.02	1.72 ± 0.18	0.57 ± 0.04	0.970	69.81 ± 0.20	53.63 ± 4.27	84.0 ± 2.31	−2.14 ± 0.10	12.2 ± 0.69	12.4 ± 0.67	100.0 ± 1.01
2	31.1 ± 0.78	0.64 ± 0.01	6.92 ± 0.01	0.23 ± 0.01	1.04 ± 0.04	0.65 ± 0.00	0.999	58.67 ± 8.99	21.78 ± 6.52	84.3 ± 1.54	2.22 ± 0.22	12.7 ± 1.66	12.9 ± 1.60	100.2 ± 2.26
3	30.7 ± 0.63	0.63 ± 0.01	6.92 ± 0.01	0.36 ± 0.02	1.42 ± 0.06	0.65 ± 0.00	0.999	153.05 ± 1.05	29.65 ± 0.36	81.8 ± 0.40	−1.79 ± 0.20	14.1 ± 1.75	14.2 ± 1.70	97.5 ± 1.91
4	31.8 ± 0.24	0.51 ± 0.01	6.74 ± 0.01	0.06 ± 0.01	0.3 ± 0.08	0.62 ± 0.06	0.999	19.32 ± 2.00	5.58 ± 1.03	86.2 ± 1.22	−3.10 ± 0.10	10.4 ± 1.12	10.8 ± 1.06	106.9 ± 2.25
5	32.6 ± 0.14	0.57 ± 0.01	6.77 ± 0.01	0.23 ± 0.01	0.8 ± 0.01	0.68 ± 0.00	0.999	58.24 ± 0.55	18.93 ± 0.76	86.0 ± 0.97	−2.60 ± 0.27	11.1 ± 0.47	11.3 ± 0.46	104.1 ± 0.52
6	32.1 ± 0.50	0.49 ± 0.01	6.74 ± 0.01	0.09 ± 0.01	0.42 ± 0.01	0.63 ± 0.01	0.999	41.21 ± 5.51	14.15 ± 0.88	82.9 ± 1.84	3.49 ± 0.03	7.69 ± 0.67	8.45 ± 0.61	114.5 ± 1.99
7	32.7 ± 0.12	0.44 ± 0.01	6.92 ± 0.01	0.06 ± 0.01	0.51 ± 0.07	0.52 ± 0.20	0.840	32.62 ± 4.61	9.5 ± 0.80	84.5 ± 2.27	3.59 ± 0.12	8.30 ± 0.81	9.06 ± 0.72	113.5 ± 2.42
8	31.8 ± 0.03	0.62 ± 0.01	6.78 ± 0.01	0.23 ± 0.01	0.91 ± 0.10	0.64 ± 0.02	0.999	110.7 ± 1.30	35.85 ± 0.15	84.9 ± 1.56	1.88 ± 0.15	13.7 ± 1.25	13.9 ± 1.21	97.9 ± 1.48
9	31.1 ± 0.13	0.49 ± 0.02	6.70 ± 0.01	0.15 ± 0.01	0.63 ± 0.01	0.63 ± 0.01	0.999	63.38 ± 3.20	18.61 ± 1.67	85.5 ± 1.90	2.12 ± 0.06	8.47 ± 1.47	8.73 ± 1.41	104.5 ± 2.49
10	31.5 ± 0.92	0.58 ± 0.00	6.70 ± 0.01	0.26 ± 0.02	1.08 ± 0.11	0.62 ± 0.02	0.990	108.7 ± 5.10	29.16 ± 3.97	85.2 ± 2.83	1.65 ± 0.19	10.1 ± 1.29	10.2 ± 1.24	99.5 ± 2.46
11	30.1 ± 0.02	0.56 ± 0.02	6.85 ± 0.01	0.21 ± 0.01	0.65 ± 0.04	0.71 ± 0.01	0.999	100.17 ± 9.74	34.99 ± 17.91	83.0 ± 3.90	1.71 ± 0.25	11.1 ± 2.11	11.2 ± 2.04	99.4 ± 3.21
12	30.4 ± 0.06	0.56 ± 0.01	6.94 ± 0.01	0.20 ± 0.01	0.56 ± 0.02	0.68 ± 0.00	0.999	65.43 ± 5.77	22.02 ± 0.03	84.0 ± 2.09	2.12 ± 0.18	10.2 ± 0.82	10.4 ± 0.78	101.9 ± 1.7
13	29.9 ± 0.11	0.42 ± 0.01	6.85 ± 0.01	0.07 ± 0.01	0.23 ± 0.01	0.65 ± 0.05	0.990	55.11 ± 1.51	17.63 ± 0.05	88.3 ± 0.51	2.35 ± 0.14	8.29 ± 0.78	8.62 ± 0.75	105.9 ± 1.67
14	30.1 ± 0.06	0.54 ± 0.01	6.80 ± 0.00	0.12 ± 0.01	0.51 ± 0.04	0.62 ± 0.01	0.990	44.17 ± 0.84	14.49 ± 0.51	85.5 ± 0.66	2.31 ± 0.17	8.87 ± 1.48	9.17 ± 1.46	104.8 ± 1.76

DM; Dry Matter. AC; Ash content; η_app_; Apparent viscosity. K; Consistency. n; Flow behavior index. G'; Storage modulus; G"; Loss modulus. Mean ± Standard Deviation.

### 2.5 Ice cream analysis

#### 2.5.1 Color analysis

The color properties of ice cream samples were determined as described in Section 2.4.2.

#### 2.5.2 Texture analysis

The ice cream samples (20 mm in height and 35 mm in diameter) were placed in standard beakers of the same brand and size and stored at −18 °C for 24 h. The hardness values were determined using the TA-TX plus instrument (Stable Micro Systems, Godalming, UK). A cylindrical probe (5 mm in diameter) was used in the analyses. The penetration depth was set at 10 mm for all measurements, and the penetration speed was 1 mm/s (Akalın et al., 2018). The hardness values were defined as the peak pressure force (g) during penetration.

#### 2.5.3 Melting behavior

The first dripping time (s) and melting rate (g/min) analyses were conducted by allowing 25 g of ice cream samples to melt under controlled conditions at room temperature (20.0 ± 2.00 °C). The samples were placed on a wire mesh with a mesh size of 0.14 mm, allowing the melted product to drip into a beaker placed underneath, thus determining the duration and the amount that dripped (Kurt et al., 2016).

#### 2.5.4 Total phenolic content

The total phenolic content of ice cream samples was determined as described in Section 2.2.

#### 2.5.5 Antioxidant activity capacity

The antioxidant activity of the ice cream samples was evaluated by measuring their scavenging capacity against the 2,2-diphenyl-1-picrylhydrazyl (DPPH) radical. To prepare the extract, a specific amount of the sample was diluted with methanol and centrifuged to obtain a clear supernatant. A 0.1 mM DPPH solution was prepared in methanol. Then, 100 μL of the sample extract was mixed with 3.9 mL of the DPPH solution. The mixture was shaken vigorously and incubated in the dark at room temperature for 30 min to allow the reaction to reach steady state. The absorbance was measured at 517 nm using a UV-VIS spectrophotometer against a methanol blank. The results were expressed as the percentage of radical inhibition (% inhibition), calculated according to the following eq. (4):

(4) $$\mathrm{Inhibition}\;\left(\%\right)=\frac{\left(A_{\mathrm{control}}-A_{\mathrm{sample}}\right)}{A_{\mathrm{control}}}\;x100$$

where A_control_ is the absorbance of the DPPH solution without the sample, and A_sample_ is the absorbance of the DPPH solution with the sample. (Demirci et al., 2024).

#### 2.5.6 Sensory analysis

Sensory evaluation was conducted using a nine-point hedonic scale as described by Lim (2011), where scores ranged from 1 (dislike extremely) to 9 (like extremely). The sensory properties of the ice cream samples, including appearance, structure (texture), taste, and overall acceptance, were evaluated by 40 untrained participants consisting of bachelor's and master's students and academic staff from the Food Engineering Department at Eskişehir Osmangazi University.

The sensory study involved non-invasive evaluation of food products and did not require formal ethical approval in accordance with institutional guidelines; therefore, no external ethics committee permission was obtained. All procedures were conducted in line with established ethical principles for research involving human participants. Participation was entirely voluntary, and appropriate measures were implemented to ensure the protection of participants' rights, privacy, and well-being. Participants were informed about the purpose and procedures of the study, potential risks (e.g., food allergies), and their right to withdraw at any time without penalty. No personally identifiable information was collected, and all data were recorded and analyzed in anonymized and aggregated form. Written informed consent was obtained from all participants prior to the sensory evaluation, confirming their voluntary participation and agreement to the use of their data for research purposes.

### 2.6 Statistical analysis

ANOVA variance analysis was performed to determine the statistical significance of the analysis results. The most suitable models for the analysis results were determined using multiple linear regression. Regression coefficients of the most suitable model and interaction terms among linear, quadratic, and cubic models were determined using Design Expert software (Stat-Ease Inc. Trial version 13.0, Minneapolis, USA). Statistical analyses were conducted with a 95% confidence interval.

## 3 Results and discussion

### 3.1 Ice cream mixes

#### 3.1.1 Physico-chemical properties

The normal pH of ice cream mix is about 6.3. Lower pH is undesirable as it increases viscosity, reduces whipping ability, impairs flavor, and results in a less stable mixture, which may lead to coagulation during processing (Acan et al., 2020; Kalicka et al., 2019; Kurt & Atalar, 2018). However, the pH values of ice cream mix samples containing different ratios of upcycled RBLW ranged from 6.70 to 6.94 (Table 3). Helstad (2018) reported that while the pH value for RBLW was 3.32, corn syrups had higher values (3.75–5.20). As expected, in our study, ice cream mixes with lower pH values were generally obtained with increased use of upcycled RBLW, although the use of stabilizers generally increased pH. Also in previous studies, using various alternative and/or novel ingredients resulted in ice cream mix pH values in a relatively wide range, as 6.28–7.26 (Akin et al., 2007; da Silva Faresin et al., 2022; Hanafi et al., 2022; Karaman et al., 2014; Nooshkam et al., 2023; Patel et al., 2006).

In this study, the linear model for the effects of independent variables revealed that stabilizer (X_2_) x glucose syrup (X_3_) and stabilizer (X_2_) x upcycled RBLW (X_1_) interactions were significant. However, the interaction between the two sugar sources was insignificant (*P* > 0.05). Additionally, a model with a relatively high *R*^*2*^ value (0.9212) was determined for the effects of independent variables on pH. These effects of upcycled RBLW (X1) on pH may alter ice cream flow, melting behavior, and textural properties. There is a relationship between the water-binding and colloidal interactions of whey proteins and pH (Genovese et al., 2022). The pH of the ice cream mixes in this study is near the neutral point of milk; localized acidity or a shift toward the isoelectric point of caseins reduces the net negative charge of the protein micelles. Mechanistically, this reduces electrostatic repulsion between micelles, potentially increasing protein-protein interactions and elevating the viscosity of the mixture, as observed in our flow behavior analysis. Therefore, when using upcycled RBLW, modification of stabilizer, fat content, and/or milk protein amounts in the ice cream composition may be necessary. An increase in ash content may reduce the stability of milk proteins (Palabiyik et al., 2023). An increase in ash content, as observed in formulations with higher RBLW, introduces additional multivalent ions, such as Ca^2^+ and Mg^2^+, into the system. These ions can act as salt bridges between negatively charged carboxyl groups on protein chains, promoting the aggregation of whey proteins and the contraction of the casein micelle. This mineral-induced bridging can influence the water-binding capacity of the proteins, thereby affecting the final texture and melting rate of the ice cream. Similarly, dry matter (DM) influences key quality parameters of ice cream mixes, such as flow behavior (Atalar et al., 2019). In this study, substituting glucose syrup with upcycled RBLW resulted in variations in these parameters. The ash content of ice cream mixes ranged from 0.42 to 0.66 g/100 g (Table 3). A significant model was determined for this parameter, with an *R*^*2*^ value of 0.7856 (Table 4). Thus, the effect of independent variables on the ash content of ice cream mixes could be defined. This result is important for the quality and stability of ice creams containing upcycled RBLW. It has been evaluated that using upcycled RBLW as a glucose syrup substitute in the ice cream mix may yield negligible results regarding the abovementioned problems. This is because it was determined that the increase in ash content varied significantly with stabilizer concentration (Supplementary Files 1 and 2). Moreover, the interactions between the independent variables were not significant (*P* > 0.05). The ash content values determined in this study are consistent with the findings of the study where licorice extract was used as a sugar and fat substitute in a mixture of protein isolate and sodium alginate (Nooshkam et al., 2023), as well as the results for samples containing persimmon puree (Karaman et al., 2014). Ahmed et al. (2023) reported that the ash content of sugar-free ice cream samples prepared with stevia from different brands ranged from 3.43 to 3.67 g/100 g. The authors emphasized a strong, positive correlation (>0.90) between the freezing point and ash content. Therefore, using upcycled RBLW as an alternative sugar source and bulking agent in ice cream production was suitable for the ash content.

**Table 4:** ANOVA results for linear and quadratic models of ice cream mix samples.

	Viscosity		Consistency		Flow Index		pH		DM		AC	
	*SS*	*P value*	*SS*	*P value*	*SS*	*P value*	*SS*	*P value*	*SS*	*P value*	*SS*	*P value*
*Model*	0.1112	0.0081	1.69	0.0406	0.0317	0.0220	0.0965	0.0003	7.09	0.2801	0.0549	0.0144
*Lineer*	0.1018	0.0015	1.36	0.0121	0.0074	0.1104	0.0889	< 0.0001	4.09	0.1712	0.0461	0.0036
*X*_*1*_*X*_*2*_	0.0080	0.1476	0.3039	0.0942	0.0044	0.0976	0.0020	0.1975	1.9	0.1891	0.0057	0.1196
*X*_*1*_*X*_*3*_	0.0010	0.5815	0.0033	0.8471	0.0014	0.3222	0.0054	0.0511	0.065	0.7973	0.0014	0.4087
*X*_*2*_*X*_*3*_	0.0011	0.5749	0.0002	0.9599	0.0041	0.1066	0.0057	0.0474	0.2279	0.6325	0.0019	0.3470
*Residual*	0.0250		0.6743		0.0100		0.0083		7.38		0.0150	
Lof*	0.0181	0.3776	0.5166	0.3066	0.0077	0.1128	0.0027	0.8933	3.97	0.6624	0.0083	0.6446
*P.E*	0.0069		0.1577		0.0023		0.0056		3.41		0.0067	
*Total*	0.1362		2.36		0.0417		0.1047		14.17		0.0699	
*R*^*2*^		0.8165	*R*^*2*^	0.7146	*R*^*2*^	0.7593	*R*^*2*^	0.9212	*R*^*2*^	0.49	*R*^*2*^	0.7856
*Adj- R*^*2*^		0.7018	*Adj- R*^*2*^	0.5362	*Adj- R*^*2*^	0.6088	*Adj- R*^*2*^	0.8719	*Adj- R*^*2*^	0.1713	*Adj- R*^*2*^	0.6517
*Pred- R*^*2*^		0.3553	*Pred- R*^*2*^	−0.1139	*Pred- R*^*2*^	0.0090	*Pred- R*^*2*^	0.7978	*Pred- R*^*2*^	−0.7721	*Pred- R*^*2*^	0.3023
*Adeq.Prec.*		7.6272	*Adeq.Prec.*	6.1459	*Adeq.Prec.*	7.8659	*Adeq.Prec.*	11.7795	*Adeq.Prec.*	4.2472	*Adeq.Prec.*	7.5397


	L^⁎^		a^⁎^		b*		Chroma		Hue Angle		G'		G"	
	*SS*	*P value*	*SS*	*P value*	*SS*	*P value*	*SS*	*P value*	*SS*	*P value*	*SS*	*P value*	*SS*	*P value*
*Model*	22.21	0.0911	4.43	0.0020	33.83	0.1279	27.43	0.1755	322.69	0.0019	35,312,61	0,2940	1594,62	0,4248
*Lineer*	7.27	0.1595	4.08	0.0003	31.76	0.0292	24.65	0.0468	315.02	0.0003	32,437,04	0,0847	1200,85	0,1858
*X*_*1*_*X*_*2*_	4.09	0.1442	0.2086	0.1454	0.2369	0.7784	0.4109	0.7055	3.33	0.4706	2124,28	0,5225	385,55	0,2798
*X*_*1*_*X*_*3*_	4.21	0.1390	0.1433	0.2181	1.03	0.5598	1.56	0.4674	4.31	0.4144	44,96	0,9249	7,18	0,8783
*X*_*2*_*X*_*3*_	2.43	0.2472	0.1433	0.2181	0.6974	0.6310	1.13	0.5334	4.54	0.4026	0,1828	0,9952	10,97	0,8499
*Residual*	12.48		0.6413		22.37		21.43		46.48		38,006,37		2295,60	
Lof*	12.23	0.0094	0.5201	0.2331	15.22	0.4477	14.58	0.4476	44.87	0.0212	32,552,11	0,1614	1708,91	0,3425
*P.E*	0.2487		0.1212		7.15		6.85		1.61		5454,27		586,69	
*Total*	34.69		5.07		56.21		48.86		369.18		73,318,98		3890,22	
*R*^*2*^		0.6402	*R*^*2*^	0.8735	*R*^*2*^	0.6019	*R*^*2*^	0.5614	*R*^*2*^	0.8741	*R*^*2*^	0,4816	*R*^*2*^	0,4099
*Adj- R*^*2*^		0.4154	*Adj- R*^*2*^	0.7945	*Adj- R*^*2*^	0.3532	*Adj- R*^*2*^	0.2872	*Adj- R*^*2*^	0.7954	*Adj- R*^*2*^	0,1576	*Adj- R*^*2*^	0,0411
*Pred- R*^*2*^		−1.1527	*Pred- R*^*2*^	0.4042	*Pred- R*^*2*^	−0.3386	*Pred- R*^*2*^	−0.4367	*Pred- R*^*2*^	0.2194	*Pred- R*^*2*^	−0,4245	*Pred- R*^*2*^	−0,6471
*Adeq.Prec.*		4.7146	*Adeq.Prec.*	9.1233	*Adeq.Prec.*	4.1627	*Adeq.Prec.*	3.7925	*Adeq.Prec.*	8.6891	*Adeq.Prec.*	37,938	*Adeq.Prec.*	33,816

DM; Dry Matter. AC; Ash content; *; Linear mixture. The test for the linear mixture terms of the Scheff polynomial model compares the linear coefficient estimates to each other rather than comparing the coefficients to zero. There is no linear effect if the linear coefficients are the same. Even though the coefficient estimates may be very large. *P* < 0.05. Adeq.Prec; Adeq. Precision.

*; Linear mixture. The test for the linear mixture terms of the Scheffe polynomial model compares the linear coefficient estimates to each other rather than comparing the coefficients to zero. There is no linear effect if the linear coefficients are the same. Even though the coefficient estimates may be very large. P < 0.05. Adeq.Prec; Adeq. Precision.

The structural stability of milk proteins is highly sensitive to the ionic environment. Mechanistically, the increased ash content from RBLW enhances ionic bridging between protein molecules, while the specific pH range of the mixture (6.70–6.94) maintains sufficient electrostatic repulsion to prevent macroscopic coagulation while allowing favorable protein-polyphenol binding. These combined effects stabilize the dairy matrix, contributing to the creamy texture and enhanced general acceptance observed in the optimized formulations.

DM value also has high interactions with other quality parameters (Acan et al., 2020). In our study, the DM values of ice cream mixes ranged from 29.9 to 32.6 g/100 g (Table 3). Although determined within a narrow range, this variation can be attributed particularly to the different DM values of glucose syrup and upcycled RBLW. Due to the negative predicted *R*^*2*^ (−0.77) for dry matter, this parameter was excluded from the predictive optimization. Corn syrups generally have approximately 80.0°Bx. In our study, the total masses of these substances were used as the independent variables, not their dry matter content. Although this approach is accepted as a limitation of the study, if the study design and modeling were to be carried out considering the dry matter of the relevant variables, the amount of water would also need to be included as an additional variable. It was based on the prediction that this situation would create disadvantages in evaluating the results. Furthermore, no significant model could be determined for the effects of independent variables and their interactions on DM values at the end of the study.

#### 3.1.2 Color

Sugars are among the components that affect the color characteristics of dairy products (McCain et al., 2018). Therefore, pigment or residual pigment levels in innovative components are critical, and it is ideal for them to have a neutral effect on color properties. In this study, the L*, a*, b*, chroma, and hue angle values of the samples ranged from 80.87 to 95.44, (−2.50) to 2.45, 9.12 to 11.49, 9.27 to 11.78, and 96.0 to 102.88, respectively (Table 3). In previous studies, Da Silva Faresin et al. (2022) determined the L*, a*, and b* values for samples prepared without any pigment source, using glucose syrup as 84.5, 4.58, and 28.3, respectively. Hanafi et al. (2022) reported these values as 78.4, −4.80, and 28.2. Variations between our findings and previous studies might be due to the origin of milk, which affects the color characteristics of ice creams. Also, using sugar alternatives (e.g., stevia) can affect the color properties of ice cream (Genovese et al., 2022). Previous studies have shown that L* and a* values are higher for ice cream mixes without glucose syrup, whereas b* values are lower (Samakradhamrongthai et al., 2021). Nooshkam et al. (2023) linked the increase in positive b* values to low sugar concentration. In this study, it was also determined that the use of upcycled RBLW resulted in a mild increase in L* value. The lack of a significant predictive model for L* (Predicted R^2^ = −1.15, Adeq. Precision <4) indicates that the lightness of the ice cream was maintained consistently across all runs. This statistically insignificant result confirms that the decolorized RBLW serves as a neutral bulking agent, successfully mimicking the visual impact of conventional glucose syrup. At the same time, stabilizer concentration had a more significant effect on other color parameters compared to other independent variables, particularly causing an increase in a* and b* values (Supplementary Files 1, 2 and 3). This was identified as a significant advantage for the potential use of upcycled RBLW in ice cream composition. Since the effects of non-pigment sources on visual properties in food technology can be negligible, the use of commercially available colorants compatible with various flavors can be enhanced (Hubbermann, 2016). Additionally, findings related to the effect of upcycled RBLW can serve as an indicator of the effectiveness of the clarification and decolorization processes. For example, upcycled RBLW obtained in powder form without these processes was characterized by a red color (Atalar et al., 2024). The main cause of these color properties is residual pigments. Previous studies have also determined that the by-products of this coloring process contain high levels of residual red-violet betaine (Nemzer et al., 2011). However, no significant models could be established for the effects of the independent variables on the ice cream mix's L*, b*, and chroma values. Important models were created for a* and hue angle, with *R*^*2*^ values of 0.8735 and 0.8741, respectively (Table 4). The interactions between the independent variables for these parameters were statistically insignificant (*P* > 0.05). These results provide critical insights into the stability of residual pigments during the processing of ice cream mixes containing upcycled RBLW. Since a* values serve as a reliable indicator for monitoring pigment stability in food matrices containing betalains (Chandran et al., 2014), they are essential for evaluating quality retention. However, it is important to note that elevated pH levels and high temperatures accelerate the degradation rate of betalains. While a pH range of 4.00–6.00 is considered optimal for the stability of these pigments, the slightly higher pH of the ice cream mix may influence the final visual characteristics (Trishitman et al., 2021).

#### 3.1.3 Flow behaviors

**Fig. 1:**
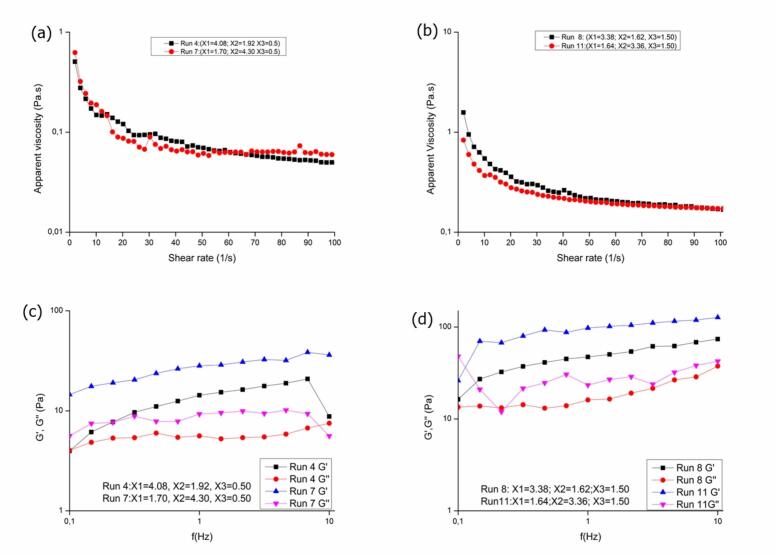
a-b: Apparent viscosity vs. shear rate for ice cream mixes c-d: Plots of storage and loss modulus as a function of frequency.

The apparent viscosity of the mixes decreased with increasing shear rate (Fig. 1a and 1b). This flow type is indicative of shear-thinning behavior, and this result is consistent with previous findings (Atalar et al., 2021; Kurt & Atalar, 2018).  The Ostwald-de Waele model was sufficient to describe the flow behavior of the ice cream mixes (*R*^*2*^ = 0.840–0.999). The independent variables' linear effects on the mixes' viscosity were significant. The *R*^*2*^ value of the model was 0.9122. At the lowest stabilizer concentration (0.50 g/100 g), increasing the upcycled RBLW (X_1_) concentration (Run 4) did not show different results compared to the low upcycled RBLW concentration (Run 7). At the highest stabilizer concentration (1.50 g/100 g), increasing upcycled RBLW concentration (Run 8) slightly enhanced viscosity compared to the low upcycled RBLW concentration (Run 11). The stabilizer concentration (X_3_) also affected the viscosity of mixes at different RBLW and glycose syrup concentrations. For example, when the upcycled RBLW concentration is constant, the increase in the stabilizer concentration (Run 11) leads to an increase in viscosity compared to the lower stabilizer concentration (Run 7). A similar result was also found for the constant glycose syrup conditions (Run 11 and 13) and constant highest stabilizer concentrations. Increasing the upcycled RBLW concentration led to an increase in consistency index from 0.65 (Run 11) to 0.91 Pa.s^n^ (Run 8) and a decrease in flow behavior from 0.71 to 0.64. The contribution of low-molecular-weight polysaccharides was related to hydration and proton-exchange properties, which indicate the solutes' affinity to interact with water via hydrogen bonding (Soukoulis & Tzia, 2018). Stabilizer concentration was also found to be significant in the constant sugar concentration. The increase in the concentration increased the consistency index and decreased the flow behavior index. The smaller n values reflect a deviation from Newtonian behavior, demonstrating the mixes' higher pseudoplasticity (Mostafavi et al., 2017). The interaction between the sugar types and stabilizer was significant for the consistency index values of mixes.  The increases in G′ and G′′ with frequency indicated the viscoelastic nature of ice cream mixtures (Fig. 1c-1d).   At higher stabilization concentrations, the highest moduli were observed, indicating that stabilizer concentration directly affected the viscoelastic properties of the ice cream mixes. The increase in glucose syrups in the formulation also increased the modulus values at both stabilizer concentration levels.  This result suggests that the network was stronger at higher glycose and stabilizer concentrations, and that the viscoelastic properties were enhanced compared to those of the other samples.  The increase in the amount of higher-molecular-weight carbohydrates in glycose syrup also contributed to the development of viscoelastic properties in ice cream mixes through their water-binding properties.  These results were also consistent with loss tangent (tan ∂ = G′′ / G′) values for the ice cream mixes obtained from the frequency sweep test.  For elastic behavior, tan ∂ < 1, whereas tan ∂ > 1 is characteristic of viscous behavior.  All the samples exhibited elastic behavior, which reflected a more structured gel-like system.

### 3.2 Ice cream

#### 3.2.1 Total phenolic content and antioxidant activity capacity

The clarified RBLW exhibited a TPC of 58.1 mg GAE/kg. While these values are lower than those of raw juice (590 mg GAE/L) (Trishitman et al., 2021) or non-decolorized powders (up to 257.4 mg GAE/kg) (Atalar et al., 2024), the retention of these compounds is critical for the functional profile of the ice cream. For the ice cream samples, these values ranged from 352.4 to 546.6 mg GAE/kg (Table 5). Based on the upcycled RBLW incorporation levels and these results, substituting glucose syrup with upcycled RBLW did not significantly alter the ice cream's phenolic profile. This is because no significant model could be established for the TPC values of the samples (*P* > 0.05). A similar trend was observed for the antioxidant activity capacity determined by the DPPH method.

**Table 5:** D-optimal mixture design for ice cream composition using clarified and decolorized red beet liquid waste (RBLW), glucose syrup and stabilizator as of independent variables. After ice cream production of the designed mixtures, measurements of various quality and composition variables were performed as model responses.

Sample	Independent Variables (g/100 g)			TPC (mg GAE/kg)	AAC (%)	Hardness (g)	Color				
	X_1_	X_2_	X_3_				L*	a*	b*	Chroma	Hue Angle
1	5.00	0.13	1.37	469.1 ± 9.75	1.93 ± 0.05	1352.0 ± 44.3	93.3 ± 0.16	1.55 ± 0.08	10.2 ± 0.62	10.4 ± 0.6	98.6 ± 0.78
2	0.30	5.00	1.20	443.6 ± 10.4	1.43 ± 0.01	2111.8 ± 105.3	90.6 ± 1.27	1.74 ± 0.04	10.3 ± 0.42	10.5 ± 0.41	99.6 ± 0.27
3	0.88	4.12	1.50	546.6 ± 10.8	1.84 ± 0.11	1948.5 ± 338.8	91.7 ± 1.20	1.51 ± 0.08	11.7 ± 0.33	11.8 ± 0.33	97.4 ± 0.19
4	4.08	1.92	0.50	400.3 ± 2.78	1.63 ± 0.11	914.1 ± 61.1	93.3 ± 1.94	2.39 ± 0.12	10.5 ± 0.76	10.7 ± 0.76	102.9 ± 0.65
5	3.03	2.52	0.95	574.3 ± 2.38	3.69 ± 0.10	1661.1 ± 256.2	92.6 ± 0.61	1.95 ± 0.04	11.2 ± 0.15	11.3 ± 0.15	99.9 ± 0.15
6	5.00	1.00	0.50	440.9 ± 12.3	3.54 ± 0.13	1331.7 ± 426.2	94.7 ± 0.94	−2.5 ± 0.08	11.0 ± 0.63	11.3 ± 0.64	102.8 ± 0.34
7	1.70	4.30	0.50	560.6 ± 28.6	3.07 ± 0.09	1850.3 ± 268.1	94.1 ± 0.89	2.45 ± 0.26	11.5 ± 1.65	11.8 ± 1.67	102.7 ± 0.48
8	3.38	1.62	1.50	488.0 ± 24.7	3.41 ± 0.38	1325.3 ± 194.9	95.4 ± 0.72	1.38 ± 0.05	11.2 ± 0.25	11.3 ± 0.24	97.0 ± 0.31
9	3.03	2.52	0.95	343.7 ± 10.3	3.38 ± 0.06	799.4 ± 85.7	88.2 ± 0.86	1.64 ± 0.04	9.12 ± 0.33	9.27 ± 0.33	100.2 ± 0.17
10	4.16	0.84	1.50	428.9 ± 32.0	4.09 ± 0.00	927.3 ± 105.7	88.0 ± 0.47	1.22 ± 0.02	9.86 ± 0.35	9.94 ± 0.35	97.1 ± 0.2
11	1.64	3.36	1.50	372.4 ± 5.83	3.95 ± 0.42	1829.0 ± 66.3	80.9 ± 0.37	1.18 ± 0.01	11.3 ± 0.21	11.3 ± 0.21	96.0 ± 0.12
12	0.30	5.00	1.20	391.5 ± 13.7	3.55 ± 0.09	2056.5 ± 266.3	85.7 ± 0.59	1.65 ± 0.04	10.8 ± 0.21	11.0 ± 0.21	98.7 ± 0.27
13	2.44	3.44	0.62	352.9 ± 5.14	4.41 ± 0.21	1638.6 ± 269.2	84.6 ± 1.55	−2.2 ± 0.07	9.83 ± 0.16	10.1 ± 0.18	101.9 ± 1.04
14	3.03	2.52	0.95	379.8 ± 1.18	4.20 ± 0.05	1998.6 ± 257.8	85.3 ± 1.21	−1.84 ± 0.1	10.8 ± 0.26	10.9 ± 0.25	99.7 ± 0.38


Sample	Independent Variables (g/100 g)			Sensory				Melting	
	X1	X2	X3	Appearance	Structure	Taste	General Acceptance	Melting rate (g/min)	FDT (s)
1	5.00	0.13	1.37	7.90 ± 0.70	6.90 ± 1.70	7.30 ± 1.19	7.40 ± 1.50	4.04 ± 0.06	832.0 ± 33.0
2	0.30	5.00	1.20	7.70 ± 0.78	6.60 ± 1.56	7.40 ± 0.92	6.80 ± 1.40	3.50 ± 0.09	787.5 ± 17.5
3	0.88	4.12	1.50	8.10 ± 0.54	6.80 ± 1.33	6.50 ± 1.02	6.90 ± 1.04	2.56 ± 0.03	508.5 ± 7.50
4	4.08	1.92	0.50	8.30 ± 0.64	7.10 ± 1.70	7.20 ± 1.83	6.80 ± 1.66	2.60 ± 0.26	420.0 ± 20.0
5	3.03	2.52	0.95	8.30 ± 0.64	7.80 ± 1.83	7.60 ± 1.80	7.80 ± 1.08	3.70 ± 0.09	797.5 ± 52.5
6	5.00	1.00	0.50	8.30 ± 0.78	6.50 ± 1.43	6.40 ± 1.50	6.80 ± 0.98	2.43 ± 0.24	781.0 ± 71.0
7	1.70	4.30	0.50	7.90 ± 1.14	6.80 ± 1.40	6.60 ± 1.62	6.40 ± 1.20	2.87 ± 0.18	723.5 ± 20.5
8	3.38	1.62	1.50	8.20 ± 0.87	7.70 ± 1.35	7.70 ± 1.42	7.90 ± 1.04	3.36 ± 0.18	745.0 ± 85.0
9	3.03	2.52	0.95	8.50 ± 0.50	7.60 ± 1.28	7.40 ± 1.36	7.60 ± 0.92	3.63 ± 0.19	695.5 ± 49.5
10	4.16	0.84	1.50	8.00 ± 0.89	7.30 ± 1.10	7.00 ± 1.41	7.30 ± 1.19	2.71 ± 0.21	606.5 ± 11.5
11	1.64	3.36	1.50	8.10 ± 1.04	8.00 ± 1.10	8.00 ± 0.77	7.80 ± 0.60	3.26 ± 0.35	740.0 ± 77.0
12	0.30	5.00	1.20	8.30 ± 0.90	7.90 ± 1.30	7.90 ± 1.22	7.95 ± 0.96	3.35 ± 0.08	758.0 ± 3.00
13	2.44	3.44	0.62	8.00 ± 0.63	7.20 ± 1.17	6.50 ± 1.12	6.55 ± 1.01	3.98 ± 0.30	720.0 ± 10.0
14	3.03	2.52	0.95	8.50 ± 0.81	7.60 ± 1.43	7.50 ± 1.43	7.70 ± 1.00	3.62 ± 0.05	816.5 ± 13.5

X_1_. X_2_. and X_3_. Clarified and Decolorized RBLW (g/100 g). Glucose Syrup (42 DE) (g/100 g). and Stabilizator (g/ 100 g) respectively.TPC; Total phenolic content; FDT; First dripping time; AAC; Antioxidant activity capacity.

X_1_. X_2_. and X_3_. Clarified and Decolorized RBLW (g/100 g). Glucose Syrup (42 DE) (g/100 g). and Stabilizator (g/ 100 g) respectively.

Furthermore, the effects of the independent variables on both parameters were negligible (Supplementary Files 1, 2 and 3). The lack of a negative sensory impact despite the presence of polyphenols can be explained by the protein-polyphenol interaction mechanism. Astringency, a common sensory drawback of high-polyphenol ingredients, is caused by the precipitation of salivary proline-rich proteins. In a dairy matrix such as ice cream, polyphenols from upcycled RBLW preferentially bind to whey proteins and caseins via hydrogen bonding and hydrophobic interactions. This masking effect prevents the polyphenols from interacting with salivary proteins, thereby neutralizing potential bitterness or astringency. Furthermore, the sugar profile of upcycled RBLW, particularly the high fructose content, may further mask phenolic notes through a cross-modal sensory interaction, in which increased sweetness perception raises the threshold for detecting bitter compounds. Therefore, substituting glucose syrup with upcycled RBLW in ice cream is not considered a limiting factor for sensory properties. This is because the interactions between whey proteins and polyphenols reduce the perception of astringency that polyphenols might cause in the mouth.

#### 3.2.2 Color

The L*, a*, b*, chroma, and hue angle values for the ice cream samples were determined to be 80.9–95.4, (−2.50)-2.45, 9.12–11.5, 9.27–11.8, 9.80–11.8, and 96.0–102.9, respectively (Table 5). It was observed that the L* values of the final product were higher than those of the ice cream mixes, and this difference was due to the freezing process. The models evaluating the effects of the independent variables on the color properties of the final ice cream samples were significant for a* and hue angle (*P* < 0.05). Consistent with the findings for the ice cream mixes, no significant models were identified for the remaining color parameters. Notably, only the interaction between upcycled RBLW (X_1_) and glucose syrup (X_2_) significantly affected the hue angle. Furthermore, the predictive power of the models for a* and hue angle improved in the final product compared to the mixes, yielding higher *R*^*2*^ values of 0.9565 and 0.9790, respectively.

#### 3.2.3 Texture

In ice cream technology, the composition and the process conditions affect textural properties. For example, fat substitutes (Velotto et al., 2021), hydrocolloids, or substances that behave like hydrocolloids affect water affinity, crystallization behavior, and structural properties, leading to the creation of ice creams with different textures (Varela et al., 2014). The sugar profile and concentration are among the compositional factors that need to be considered (Jardines et al., 2020). Texture even influences the perception of sweetness (McCain et al., 2018). Additionally, there is a strong relationship between ice cream hardness and firmness and its structural properties; for example, the geometry and morphology of air cells, as well as their interactions with components, especially proteins and fats, affect the final product's textural properties (Saremnezhad et al., 2020). Furthermore, it has been reported that the effects of sugar substitutes on freezing point are related to texture (da Silva Faresin et al., 2022). In our study, the hardness values of the ice cream were lower than those reported by Karaman et al. (2014) and Ahmed et al. (2023). The main reason for variability in results across studies is compositional differences. Furthermore, although a significant model was established for the linear relationship between the independent variables and hardness values, the interaction between them was not significant (*p* > 0.05). The *R*^*2*^ value for the linear model was relatively low at 0.5858 (Table 6). The results confirm that upcycling RBLW into ice cream is technologically advantageous for texture, as it yields a creamy, soft mouthfeel without requiring complex ingredient interactions. However, the relatively low *R*^*2*^ suggests that for future commercial scaling, the freezing process (overrun) should be more tightly controlled to improve the predictability of the final texture. It was anticipated that the independent variables would directly impact textural properties by altering the stabilizer and sugar profiles. This expectation stems from the significant differences between the sugar profiles of conventional glucose syrup and the upcycled RBLW (Section 2.3). Specifically, the RBLW composition contains a higher concentration of monosaccharides, particularly fructose. Fructose is characterized by its high moisture affinity, which influences the formulation's water-binding properties (Ergun et al., 2010). Given that interactions exist between the water-binding capacity of ingredients and the final texture of ice cream, these compositional shifts are critical to product performance (Genovese et al., 2022). Ahmed et al. (2023) also found a positive correlation between instrumental hardness and glucose and fructose concentrations. Therefore, a decrease in hardness is expected when using upcycled RBLW. Glucose syrup resulted in lower hardness than upcycled RBLW in the ice cream samples (Supplementary Files 2 and 3). However, the change in stabilizer concentration had a negligible effect on hardness. DM values should also be considered when evaluating these effects. Using upcycled RBLW instead of glucose syrup at an equivalent mass result in a lower TSS in the ice cream. Ice creams with lower TSS have a greater capacity to freeze water, which may result in more ice crystals and affect texture (Hanafi et al., 2022). The increase in crystallization also raises hardness (Nooshkam et al., 2023). Despite this interaction, using upcycled RBLW (X_1_) resulted in a softer texture, a remarkable finding. Additionally, during consumption, tongue movement, melting behavior, and in-mouth textural perception significantly influence perceptions of product quality. It is generally accepted that good ice cream exhibits a creamy, soft, and foamy texture, and that this texture should be uniform (Genovese et al., 2022). (See Table 7.)

**Table 6:** ANOVA results for linear and quadratic models of ice cream samples.

	L*		a*		b*		Chroma		Hue Angle	
	*SS*	*P value*	*SS*	*P value*	*SS*	*P value*	*SS*	*P value*	*SS*	*P value*
*Model*	78.14	0.6489	2.41	<0.0001	2.30	0.5723	2.34	0.5654	67.71	< 0.0001
*Lineer*	43.75	0.4239	2.34	<0.0001	0.5778	0.6184	0.5089	0.6543	66.21	< 0.0001
*X*_*1*_*X*_*2*_	23.06	0.3445	0.0469	0.1020	0.0202	0.8549	0.0088	0.9043	1.47	0.0217
*X*_*1*_*X*_*3*_	11.41	0.4999	0.0217	0.2444	1.46	0.1471	1.59	0.1327	0.0081	0.8382
*X*_*2*_*X*_*3*_	12.11	0.4874	0.0185	0.2797	1.18	0.1867	1.31	0.1680	0.0012	0.9377
*Residual*	182.80		0.1099		4.53		4.55		1.45	
Lof*	144.00	0.2711	0.0592	0.6610	2.06	0.7673	2.09	0.7628	0.8818	0.5615
*P.E*	38.80		0.0507		2.47		2.46		0.5692	
*Total*	260.94		2.52		6.82		6.89		69.16	
*R*^*2*^		0.2994	*R*^*2*^	0.9565	*R*^*2*^	0.3368	*R*^*2*^	0.3401	*R*^*2*^	0.9790
*Adj- R*^*2*^		−0.1384	*Adj- R*^*2*^	0.9292	*Adj- R*^*2*^	−0.0777	*Adj- R*^*2*^	−0.0723	*Adj- R*^*2*^	0.9659
*Pred- R*^*2*^		−1.0313	*Pred- R*^*2*^	0.8863	*Pred- R*^*2*^	−1.3372	*Pred- R*^*2*^	−1.3216	*Pred- R*^*2*^	0.9335
*Adeq.Prec.*		2.3668	*Adeq.Prec.*	15.7858	*Adeq.Prec.*	2.9777	*Adeq.Prec.*	2.9064	*Adeq.Prec.*	22.9514


	Appearance		Structure		Taste		General Acceptance	
	*SS*	*P value*	*SS*	*P value*	*SS*	*P value*	*SS*	*P value*
*Model*	0.4600	0.0884	1.82	0.1831	1.70	0.3383	2.52	0.0743
*Lineer*	0.0664	0.3963	0.3891	0.3871	0.8776	0.2380	1.32	0.0596
*X*_*1*_*X*_*2*_	0.1733	0.0480	1.05	0.0430	0.1026	0.5428	0.2737	0.2289
*X*_*1*_*X*_*3*_	0.0968	0.1196	0.3705	0.1911	0.7212	0.1305	0.8726	0.0485
*X*_*2*_*X*_*3*_	0.1464	0.0644	0.3564	0.1989	0.7309	0.1283	0.9463	0.0417
*Residual*	0.2550		1.45		2.03		1.29	
Lof*	0.0483	0.9704	0.5814	0.8260	1.89	0.0606	0.6091	0.7468
*P.E*	0.2067		0.8717		0.1450		0.6813	
*Total*	0.7150		3.27		3.74		3.81	
*R*^*2*^		0.6434	*R*^*2*^	0.5554	*R*^*2*^	0.4561	*R*^*2*^	0.6611
*Adj- R*^*2*^		0.4205	*Adj- R*^*2*^	0.2776	*Adj- R*^*2*^	0.1161	*Adj- R*^*2*^	0.4492
*Pred- R*^*2*^		−0.1174	*Pred- R*^*2*^	−0.3534	*Pred- R*^*2*^	−0.5634	*Pred- R*^*2*^	−0.0452
*Adeq.Prec.*		4.9532	*Adeq.Prec.*	4.2755	*Adeq.Prec.*	3.0441	*Adeq.Prec.*	5.4529


	TPC		AAC		Hardness		First Dropping Time		Melting Rate	
	*SS*	*P value*	*SS*	*P value*	*SS*	*P value*	*SS*	*P value*	*SS*	*P value*
*Model*	8192.00	0.9568	4.00	0.6501	1.531E+06	0.1457	67,447.77	0.5164	2.47	0.0740
*Lineer*	607.85	0.9650	0.2308	0.9075	1.458E+06	0.0329	778.98	0.9740	0.0754	0.7930
*X*_*1*_*X*_*2*_	831.64	0.7624	3.00	0.1484	58,423.69	0.5295	8210.09	0.4766	0.0190	0.7373
*X*_*1*_*X*_*3*_	4228.33	0.5005	0.2855	0.6352	11,271.24	0.7802	50,931.36	0.1000	1.83	0.0093
*X*_*2*_*X*_*3*_	5499.51	0.4443	0.1826	0.7036	11,776.91	0.7755	41,140.35	0.1332	1.41	0.0174
*Residual*	67,968.88		9.39		1.082E+06		1.178E+05	0.5164	1.26	0.0740
Lof*	35,826.47	0.6768	6.81	0.3741	3.159E+05	0.9167	1.089E+05	0.0658	1.25	0,0044
*P.E*	32,142.41		2.58		7.664E+05		8903.79		0.0151	
*Total*	76,160.88		13.40		2.613E+06		1.853E+05		3.73	
*R*^*2*^		0.1076	*R*^*2*^	0.2989	*R*^*2*^	0.5858	*R*^*2*^	0.3641	*R*^*2*^	0.6614
*Adj- R*^*2*^		−0.4502	*Adj- R*^*2*^	−0.1393	*Adj- R*^*2*^	0.3270	*Adj- R*^*2*^	−0.0334	*Adj- R*^*2*^	0.4498
*Pred- R*^*2*^		−1.9949	*Pred- R*^*2*^	−2.0071	*Pred- R*^*2*^	−0.1487	*Pred- R*^*2*^	−1.7738	*Pred- R*^*2*^	−0.2992
*Adeq.Prec.*		1.4059	*Adeq.Prec.*	2.0870	*Adeq.Prec.*	4.2289	*Adeq.Prec.*	2.6132	*Adeq.Prec.*	5.0919

*; Linear mixture. The test for the linear mixture terms of the Schffe polynomial model compares the linear coefficient estimates to each other rather than comparing the coefficients to zero. There is no linear effect if the coefficients are equal. Even though the coefficient estimates may be very large. P < 0.05. Adeq.Prec; Adeq. Precision.

*; Linear mixture. The test for the linear mixture terms of the Schffe polynomial model compares the linear coefficient estimates to each other rather than comparing the coefficients to zero. There is no linear effect if the linear coefficients are the same. Even though the coefficient estimates may be very large. P < 0.05. Adeq.Prec; Adeq. Precision.

^⁎^; Linear mixture. The test for the linear mixture terms of the Schffe polynomial model compares the linear coefficient estimates to each other rather than comparing the coefficients to zero. There is no linear effect if the linear coefficients are the same. Even though the coefficient estimates may be very large. P < 0.05. Adeq.Prec; Adeq. Precision.

**Table 7:** Optimum conditions and experimental validation of the clarified and decolorized RBLW-based formulation.

Factor A	Factor B	Factor C	Response 1	Response 2	Response 3	Response 4
Clarified and Decolorized RBLW (g/100 g)	Glucose Syrup (g/100 g)	Stabilizator (g/100 g)	Consistency Index (Pa.s^n^)	First Dripping Time (s)	Taste	General Acceptance
3.68	1.69	1.12	1.31	785	7.62	7.84

**Experimental Results**						
	**Predicted (Optimum)**		**Observed (Actual)**		**% Deviation**	
Consistency Index (Pa.s^n^)	1.31		1.25		−4.58	
First Dripping Time (s)	785		745		−5.09	
Taste	7.62		7.72		+1.31	
General Acceptance	7.84		7.83		−0.13	

#### 3.2.4 Sensory properties

**Fig. 2:**
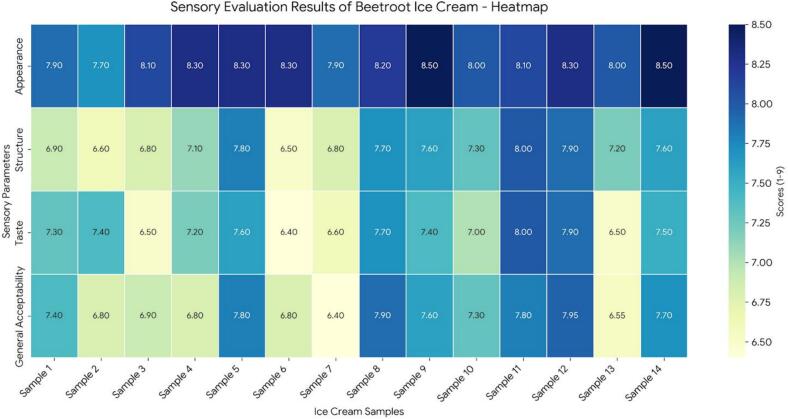
Heatmap of sensory evaluation results for beetroot ice cream formulations (Samples 1–14). The color gradient represents hedonic scores ranging from 1 to 9 across four parameters: Appearance, Structure, Taste, and General Acceptability.

In this study, the sensory attributes of the ice cream samples, including appearance, structure, taste, and general acceptance, were evaluated by panelists using a 9-point scale (Fig. 2). Achieving scores of 6.40 or higher for all sensory attributes was considered promising for the use of innovative raw materials (Table 5). The fact that the appearance (7.70–8.50) was rated higher than other sensory parameters can be considered a positive finding, considering the decolorization and clarification processes applied to upcycled RBLW (Table 5). Additionally, the increase in appearance scores with increasing upcycled RBLW (X_1_) usage and the negative effect of stabilizer (X_3_) usage on this increase were noteworthy findings. A negative relationship was observed between glucose syrup and stabilizer concentrations (Fig. 2). Although the preference level for the ice cream sample structure (6.50–7.80) was lower than for other attributes, positive results were still obtained (Table 5). The effect of the stabilizer amount, rather than the sugar sources, was observed on this parameter. There are strong relationships between texture, structural properties, and preference (Nooshkam et al., 2023). The independent variables were also found to influence ice cream hardness, the main texture parameter. This effect may also explain the differences observed in sensory evaluation. Panelists were scored taste of the samples between 6.40 and 8.00 (Table 5). Aside from their impact on sweetness, sweeteners are also important in the development of retronasal olfaction during oral processing (Soukoulis & Tzia, 2018). Some hydrocolloids, including polysaccharides, can also exhibit aroma-masking effects (Varela et al., 2014). However, studies have reached different conclusions on this matter. For example, Soukoulis et al. (2008) reported that the presence of hydrocolloids increased perceived aroma intensity. Using volume agents with higher sweetness levels can enhance aroma intensity, while macromolecular sweeteners can play an aroma-suppressing role (Soukoulis et al., 2010). The use of certain high-intensity sweeteners and various polyols as sugar substitutes in ice cream has been reported to result in adverse outcomes, depending on the usage rate, due to changes in aftertaste characteristics (Genovese et al., 2022). However, in this study, replacing glucose syrup with upcycled RBLW did not cause flavor issues in the ice cream. Additionally, when examining the results for general acceptance (6.40–7.95), the effects of increasing upcycled RBLW and stabilizer concentrations were another noteworthy, positive finding. One limitation of this study was the insufficient lab-scale equipment for sample preparation, leading to an overrun. This could have affected some structural characteristics of the samples and, therefore, the structure parameter in sensory evaluation. Further studies to obtain and analyze ice cream samples with higher overrun would significantly advance the assessment of the potential of upcycled RBLW for use in ice cream technology.

#### 3.2.5 Melting behavior

The initial dripping times and melting rates were 420.0–832.0 s and 2.56–4.04 g/min, respectively. No significant model was identified for the change in melting behavior due to the effect of independent variables in the samples (*P* > 0.05) (Table 6). However, it has been emphasized that ice creams with higher hardness melt more slowly. Slow and uniform melting behavior is among the desired characteristics of ice cream (Acan et al., 2020). Previous studies have identified an inverse relationship between melting rate and texture properties (Nooshkam et al., 2023). Since using upcycled RBLW (X_1_) decreases ice cream hardness, it can be expected to accelerate the melting. The increase in the amount of frozen water in ice cream, the low solubility of the sweeteners used, and the relatively slow melting of amorphous solids by sugars during freezing may reduce ice cream's melting rate (Moriano & Alamprese, 2017). The saccharides in the upcycled RBLW composition, due to their lower molecular weight compared to glucose syrup, may lower the freezing point, thus affecting the melting rate (Helstad, 2018). Additionally, Varela et al. (2014) stated that the viscosity of the ice cream mix shows a strong correlation with a decrease in melting rate and an increase in shape retention. In our study, there were also apparent viscosity effects of the independent variables and their interactions, and these conditions and changes have the potential to improve ice cream quality.

#### 3.2.6 Optimization and model validation

The optimization study using Response Surface Methodology (RSM) identified the ideal formulation for maximizing product quality as 3.68 g/100 g of clarified and decolorized RBLW, 1.69 g/100 g of glucose syrup, and 1.12 g/100 g of gelatin, based on the maximum consistency index, first dripping time, first dripping time and general acceptance score. To verify the reliability of the developed models, validation experiments were conducted under these optimal conditions. The experimental (observed) results showed strong alignment with the model-predicted values, yielding a consistency index of 1.25 Pa.s^n^, a first dripping time of 745 s, a taste score of 7.72, and a general acceptance score of 7.83. The percentage deviation between the predicted and observed values remained remarkably low, ranging from −5.09% to +1.31%. These results demonstrate the high predictive capacity of the mathematical models and confirm that RBLW-based formulations can be accurately optimized to meet both technological and sensory quality standards in ice cream production.

## 4 Conclusion

This study demonstrates that industrial red beet colorant process liquid waste (RBLW) can be successfully upcycled into a sustainable glucose syrup alternative for ice cream production through a series of clarification, decolorization, and concentration processes. The treatment process, involving ion-exchange and adsorber resins, effectively increased the clarity (T_625_) to 77.3%. The resulting upcycled RBLW proved to be a viable functional ingredient, as significant models (*R*^2^ = 0.60–0.92) were established for the physicochemical and rheological properties of the ice cream mixes. The pH values of the ice cream mixes remained within a stable range of 6.70 to 6.94, while the ash content ranged from 0.42 to 0.66 g/100 g. The dry matter (DM) of the mixes ranged from 29.9 to 32.6 g/100 g. The use of upcycled RBLW increased the consistency index from 0.65 to 0.91 Pa.s^n^ and enhanced the viscosity of the mixes at higher stabilizer concentrations. All samples exhibited elastic behavior (tan δ < 1), indicating a well-structured gel-like system. Despite the origin of the waste, the resin treatments ensured high visual quality, with appearance scores ranging from 7.70 to 8.50. General acceptance scores were also high, reaching up to 7.95. The ideal formulation was identified as 3.68 g/100 g RBLW, 1.69 g/100 g glucose syrup, and 1.12 g/100 g stabilizer. Validation experiments confirmed the model's accuracy, showing a low deviation of −5.09% to +1.31% from predicted values. In conclusion, upcycled RBLW can replace conventional glucose syrup without compromising the technological or sensory attributes of ice cream. This approach supports circular economy principles by transforming industrial side streams into value-added food ingredients. Future research should focus on process scale-up and shelf-life assessment to fully realize the commercial potential of this sustainable alternative.

## CRediT authorship contribution statement

**Tahra ElObeid:** Writing – review & editing, Validation, Methodology, Conceptualization. **İlyas Atalar:** Writing – review & editing, Methodology, Conceptualization. **Burcu Tüzün:** Formal analysis. **Ibrahim Palabiyik:** Writing – review & editing, Writing – original draft. **Omer Said Toker:** Writing – review & editing. **Nevzat Konar:** Writing – review & editing, Methodology, Conceptualization.

## Informed consent

Not applicable.

## Institutional review board statement

Not applicable.

## Funding

This study was granted by TUBİTAK (Project No: 123O211). “Qatar National Library funded the publication of this article.”

## Declaration of competing interest

The authors declare that they have no known competing financial interests or personal relationships that could have appeared to influence the work reported in this paper.

## Data Availability

Data will be made available on request.
