# Supplementary material for: Upcycling clarified and decolorized red beet waste into a sustainable glucose syrup alternative for ice cream production
Source: Food Chem X. 2026 Jun 6;37:104073. doi: 10.1016/j.fochx.2026.104073 (PMC13260206; doi:10.1016/j.fochx.2026.104073)

**Supplementary File 1**. The interaction effects of clarified and decolorized red beet liquid waste (RBLW) and stabilizer on dependent variables for ice cream samples prepared according to D-optimal mixture design


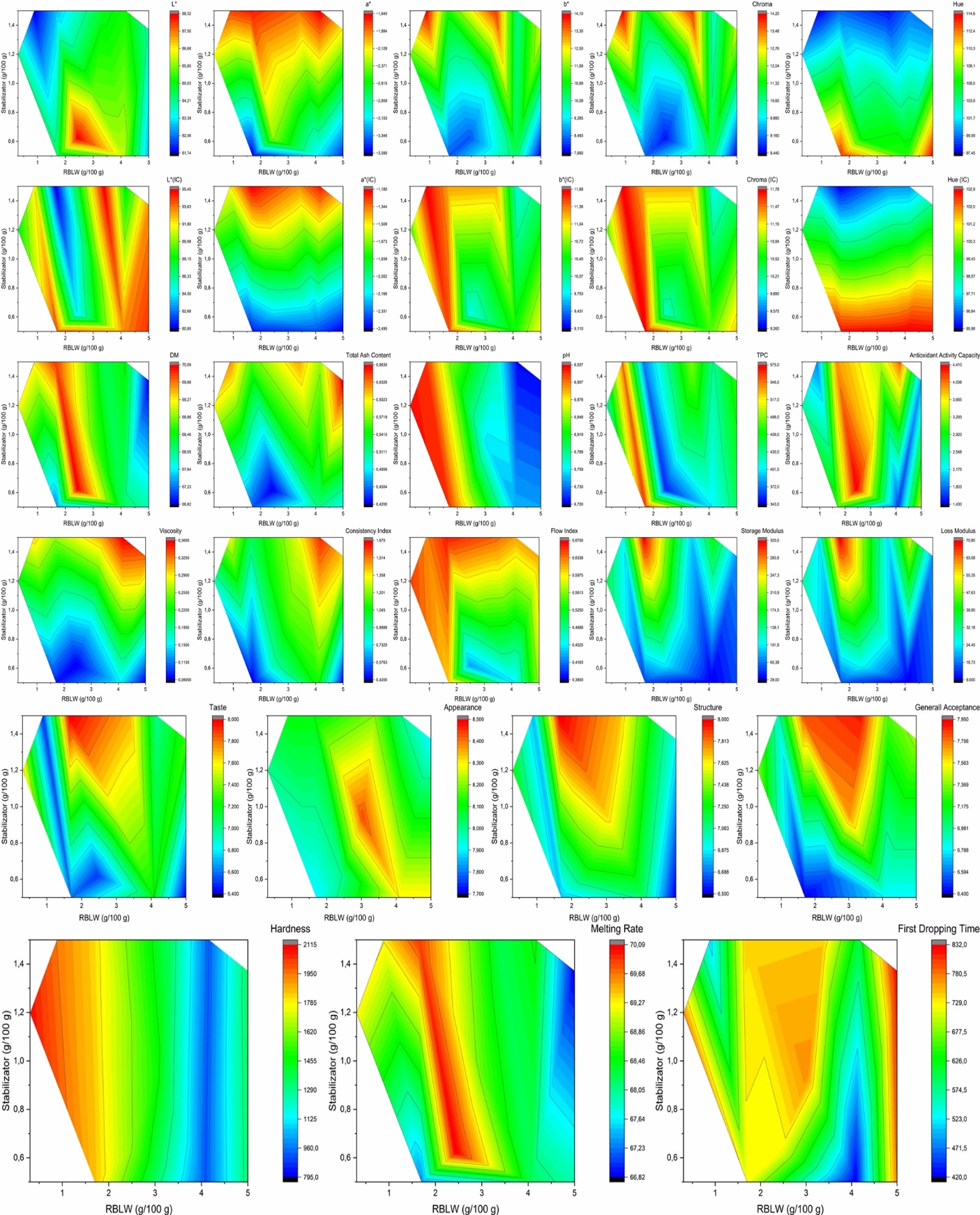


**Supplementary File 2.** The interaction effects of stabilizer and glucose syrup on dependent variables for ice cream samples prepared according to D-optimal mixture design


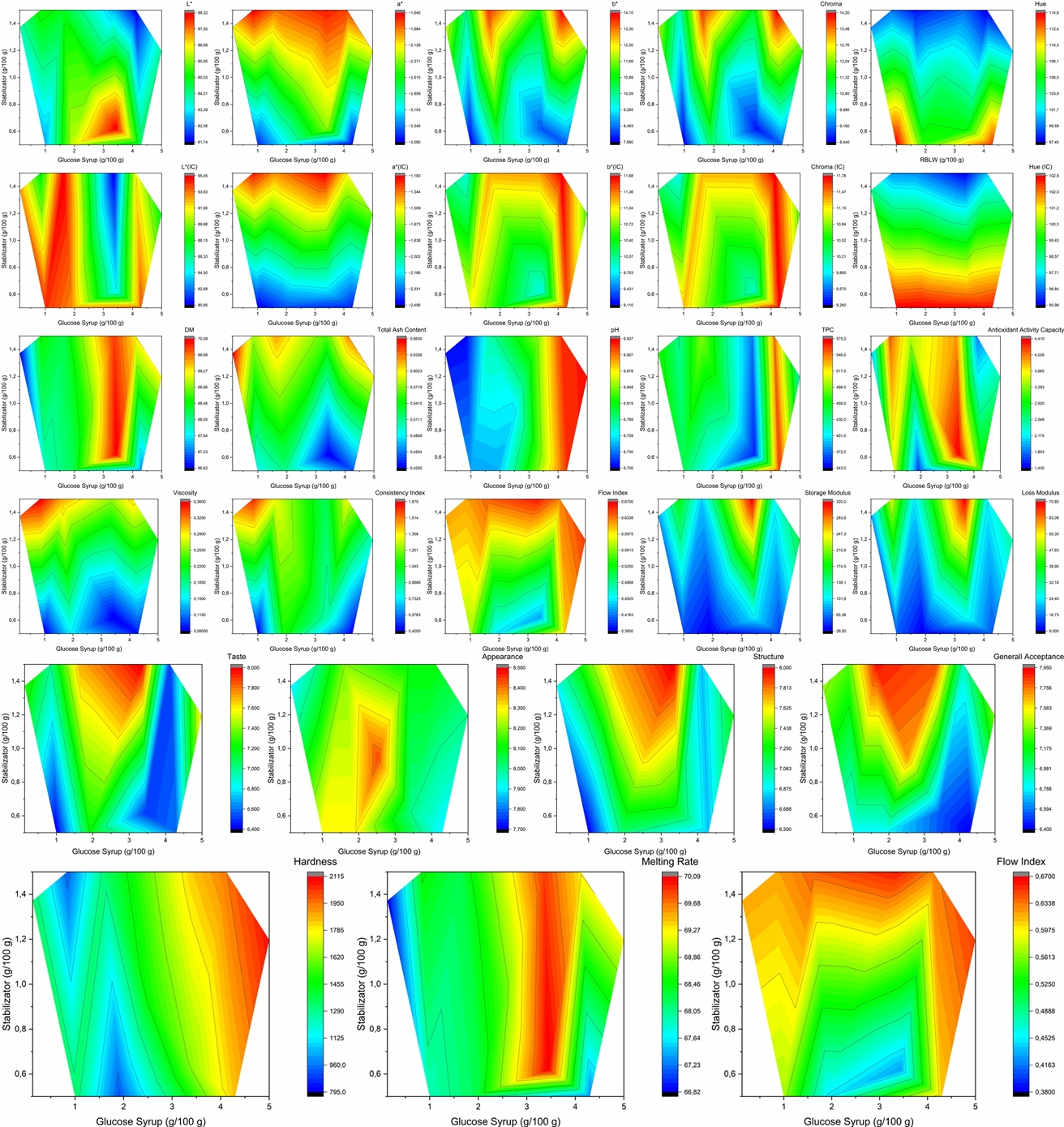


**Supplementary File 3.** The interaction effects of clarified and decolorized red beet liquid waste (RBLW) and glucose syrup on dependent variables for ice cream samples prepared according to D-optimal mixture design


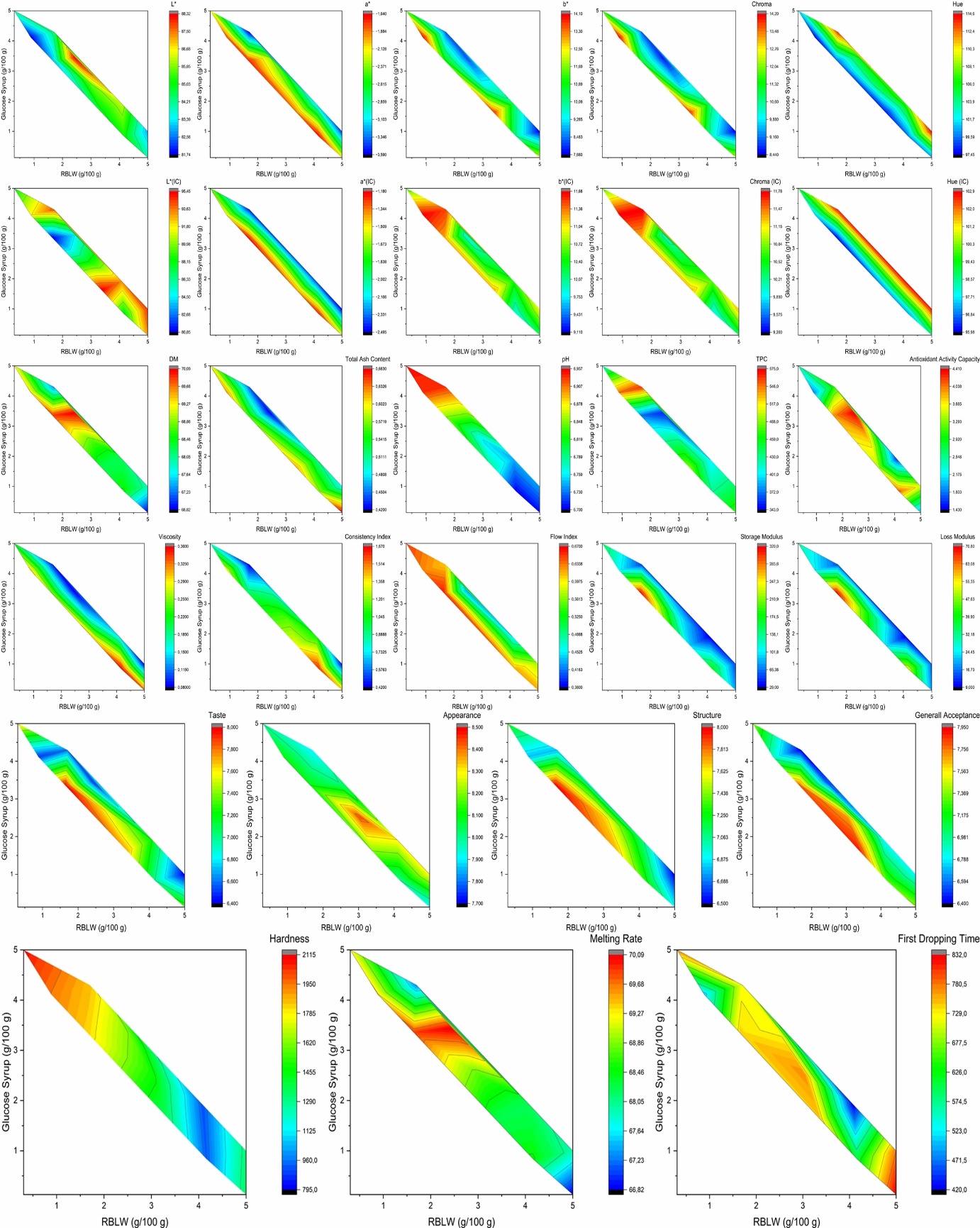

Supplement: Supplementary material 1 [file mmc1.docx]
