# Supplementary material for: Upcycling clarified and decolorized red beet waste into a sustainable glucose syrup alternative for ice cream production
Source: Food Chem X. 2026 Jun 6;37:104073. doi: 10.1016/j.fochx.2026.104073 (PMC13260206; doi:10.1016/j.fochx.2026.104073)
Supplement: Supplementary material 2 [file mmc2.docx]

**INFORMED CONSENT FORM FOR SENSORY EVALUATION PANELISTS TO PARTICIPATE IN:**

**Upcycling Clarified and Decolorized Red Beet Waste into a Sustainable Glucose Syrup Alternative for Ice Cream Production**

You are cordially invited to participate in a sensory evaluation study for various ice cream samples. This research is conducted as part of the TÜBİTAK-funded project (Project No: 123O211) titled: "Production of Deionized Sugar as a Glucose Syrup Alternative from Liquid Wastes of Natural Colorant Processes and Determination of Its Usage Possibilities in Model Foods." The primary objective of this research is to assess consumer preference and acceptance of different ice cream formulations. These products will be evaluated using a 9-point Hedonic Scale, as described by Lim (2011). Unlike descriptive analysis, which requires intense training, this method relies on your personal preference as a consumer. You will be asked to taste and rate the samples based on the following attributes:

- Appearance
- Structure (Texture)
- Taste
- General Acceptance

Ratings will be classified on a scale from 1 (Dislike Extremely) to 9 (Like Extremely).

This study is open to bachelor's, master's, and academicians in the Food Engineering Department at Eskisehir Osmangazi University. No prior sensory training is required. The samples contain dairy. If you have an existing allergy to milk, dairy products, or any common ice cream ingredients, or if you experience an allergic reaction during the study, you must discontinue participation immediately. Your participation is entirely voluntary, and no monetary compensation will be provided. You may withdraw from the study at any time and for any reason without penalty. The study will be conducted in the laboratories of the Food Engineering Department at Eskisehir Osmangazi University.

Your individual data and performance in this research will remain strictly confidential. Responses are coded, and any subsequent publications or presentations of the results will only include aggregated group data. No identifiable personal information will be disclosed.

I understand the above information and voluntarily consent to participate in the study described above. I have been given a copy of this consent form.

| **Participant name** | **Signature** | **Date** |
| --- | --- | --- |
|  |  |  |
|  |  |  |
|  |  |  |
|  |  |  |
|  |  |  |
|  |  |  |
|  |  |  |
